# Three Consecutive Days of Interval Runs to Exhaustion Affects Lymphocyte Subset Apoptosis and Migration

**DOI:** 10.1155/2014/694801

**Published:** 2014-05-08

**Authors:** James W. Navalta, Ramires Alsamir Tibana, Elizabeth A. Fedor, Amilton Vieira, Jonato Prestes

**Affiliations:** ^1^Kinesiology and Nutrition Sciences, University of Nevada, Las Vegas, 4505 S. Maryland Parkway, P.O. Box 453034, Las Vegas, NV 89154-3034, USA; ^2^Graduation Program on Physical Education, Catholic University of Brasilia, 71966-700 Brasilia, DF, Brazil; ^3^Department of Kinesiology, Recreation and Sport, Western Kentucky University, Bowling Green, KY 42101-1089, USA; ^4^College of Physical Education, University of Brasilia, 72-910-910 Brasilia, DF, Brazil

## Abstract

This investigation assessed the lymphocyte subset response to three days of intermittent run exercise to exhaustion. Twelve healthy college-aged males (*n* = 8) and females (*n* = 4) (age = 26 ± 4 years; height = 170.2 ± 10 cm; body mass = 75 ± 18 kg) completed an exertion test (maximal running speed and VO_2max_) and later performed three consecutive days of an intermittent run protocol to exhaustion (30 sec at maximal running speed and 30 sec at half of the maximal running speed). Blood was collected before exercise (PRE) and immediately following the treadmill bout (POST) each day. When the absolute change from baseline was evaluated (i. e., Δ baseline), a significant change in CD4+ and CD8+ for CX3CR1 cells was observed by completion of the third day. Significant changes in both apoptosis and migration were observed following two consecutive days in CD19+ lymphocytes, and the influence of apoptosis persisted following the third day. Given these lymphocyte responses, it is recommended that a rest day be incorporated following two consecutive days of a high-intensity intermittent run program to minimize immune cell modulations and reduce potential susceptibility.

## 1. Introduction

One commonly cited barrier to exercise is a lack of time [1], which has increased the interest of practitioners and researchers for investigating the efficacy of high-intensity interval training (HIT) as a viable alternative to traditional endurance-based exercise. The inclusion of HIT as part of an exercise program could offer a more time-efficient approach to achieve specific performance and health goals. HIT training requires individuals to perform brief periods of high-intensity exercise (typically >90% VO_2max_) separated by recovery periods of lower-intensity aerobic exercise or rest [2]. Previous investigations have reported that running at intensity equal to maximal oxygen uptake or at maximal running velocity (V_max⁡_) may lead to superior chronic adaptations when compared to continuous running training for high-level athletes [3] or elicit rapid improvement in the “aerobic fitness” of recreational athletes [4], as well as controlling blood pressure and improving endothelial function [5, 6].

Although HIT has been widely used and recommended as part of a training program, evidence has shown that intense exercise triggers the apoptosis of lymphocytes, which may in part account for exercise-induced decrease in the count of circulating lymphocytes (lymphocytopenia) and reduced immunity [7]. Furthermore, data from our research group revealed that exercise intensities greater than 76% of VO_2max_ displayed an incremental increase in the percentage of apoptotic lymphocytes culminating with the greatest values observed at maximal exertion [8]. To note, annexin V (member of the annexin family of intracellular proteins) that binds to phosphatidylserine has been widely used to identify apoptotic cells [9]. Nevertheless, the movement of cells from the circulation (migration) is another possible contributing mechanism to postexercise lymphocytopenia [10]. This migration process may be identified by the expression of CX3CR1 (CX3C chemokine receptor 1), since the interaction of CX3-CR1 with its ligand mediates cell adhesion and migration [11].

The frequency, intensity, and duration of exercise and preexercise fitness level are all important determinants of the response to exercise [12]. Tuan et al. [13] submitted trained runners to treadmill exercise at 85% VO_2max_ for 30 minutes daily over three consecutive days and found that high-intensity exercise induced a significant dysfunction of the mitochondrial energy status in peripheral blood immune cells, which was accompanied by an increased propensity for apoptosis and an increase in tumor necrosis factor-alpha. However, based on the highly specific nature of immune cells, it would be expected that each lymphocyte subset may respond to exercise in a different manner and that subfractions display a differing lymphocytopenia response in the postexercise period.

To the best of our knowledge, this is the first study to investigate cell markers of apoptosis and migration following three days of consecutive high-intensity interval treadmill running in different lymphocytes' subsets. Thus, the aim of the present study was to investigate the effects of three consecutive days of high-intensity interval running on markers of apoptosis and migration in CD4+ (helper) and CD8+ (cytotoxic) T cells and CD19+ (B-cells) in untrained individuals. We hypothesized that on the third day of high-intensity interval running, cell surface markers of apoptosis and migration would be significantly increased as compared with previous days.

## 2. Methods

### 2.1. Subjects

Twelve college-aged subjects, males (*n* = 8) and females (*n* = 4) (26 ± 4 years, 75 ± 18 kg, and 170 ± 10 cm), volunteered to participate in this study. Males and females were included in the cohort because previous observations from our laboratory have found that neither gender nor menstrual cycle phase influences exercise-induced lymphocyte apoptosis in untrained subjects [14]. The subjects were healthy and without orthopedic or cardiovascular diseases. All participants completed a health screening questionnaire prior to beginning any exercise [15]. Before taking part in the experiment, volunteers were informed of all procedures, purposes, benefits, and risks of the study and signed a written informed consent. The participants were asked to refrain from any form of training or vigorous physical activity during the day before the onset of the study and were instructed not to take any anti-inflammatory agents, steroids, or antioxidant supplements before or during the study period. In addition, they were instructed to get a good night rest, be well hydrated, and avoid caffeine and alcohol for eight hours prior to testing. The investigation was approved by the Institutional Ethics Committee in accordance with the Helsinki Declaration.

### 2.2. Exercise and Testing Protocols

**Figure 1:**
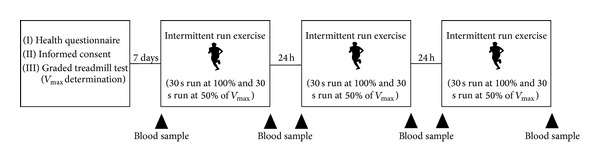
Graphical description of study timeline.

An overview of the experimental protocol is presented in Figure 1. All participants reported to the laboratory on four separate days. On the first day a graded treadmill test protocol was completed to determine maximal running velocity (V_max⁡_). The initial velocity was set at 10 km·h^−1^ and was increased by 1 km·h^−1^ every 2 min. The V_max⁡_ was defined as the highest running velocity maintained for more than 1 minute. If the velocity at fatigue was only maintained for 1 minute (half of the stage duration), then V_max⁡_ was considered to be equal to the velocity during the previous stage plus half the velocity increase between the last stages [16].

During the following week participants reported to the laboratory on consecutive three days to complete intermittent treadmill runs exercise to exhaustion. The intermittent exercise consisted of alternating 30-second runs at 100% and 30-second runs at 50% of V_max⁡_. Billat et al. [16] previously demonstrated that this intermittent protocol allowed subjects to maintain maximal oxygen uptake for longer than continuous slower running and was tolerated well by untrained individuals. All testing and experimental sessions were completed between 9:00 and 12:00 a.m. to avoid any confounding effect of the circadian rhythm.

### 2.3. Blood Analysis

Blood samples for lymphocyte subset apoptosis and migration determination were collected before (PRE) and immediately following the treadmill bouts (POST) for the three consecutive days of intermittent running. All antibodies and reagents were obtained from eBioscience (San Diego, CA, USA) unless otherwise noted. The lymphocyte subsets were CD4+, CD8+, and CD19+ with cell surface markers being annexin V and CX3CR1 (BioLegend, San Diego, CA, USA). The blood analysis protocol for the determination of lymphocyte subsets has been described elsewhere [17]. Whole blood (20 µL) was added to an appropriate antibody panel (250 µL) and incubated in a dark room for 30 min. After incubation, samples were centrifuged for 5–10 min, decanted, and thoroughly vortexed before the addition of red blood cell lysis buffer. After the 10 min lysis period, phosphate buffered saline was added and samples were centrifuged, decanted, and vortexed before analysis by flow cytometry (C6, Accuri, Ann Arbor, MI, USA). Cell apoptosis and migration were determined through the use of annexin V and CX3CR1, respectively. At least 10,000 events were counted in the lymphocyte gate, initially determined from front and side-scatter characteristics. Further gating to distinguish CD4+, CD8+, and CD19+ populations was determined in the fluorescence channel (FL-2) via the phycoerythrin (PE) fluorochrome.

### 2.4. Statistical Analysis

The Shapiro-Wilk normality test and homoscedasticity test were used to determine normal distribution of the data. Absolute changes from rest (Δ baseline) values were calculated according to the following formula: [(measure-baseline)•baseline^−1^]•100. As we expected for the absolute change from rest with regard to apoptotic and migratory markers to be similar to the change in cell counts, the Chi squared test (*χ* ^2^) was applied. Based on previous investigation [7], a large effect size was anticipated, but to use a conservative approach, sample size was calculated using a medium effect size corresponding to a 25% relative increase in postexercise apoptosis. To detect this increase in lymphocyte apoptosis, it was determined that a minimum of at least eight subjects was necessary. Statistical analyses were performed using SPSS 20.0 (SPSS, Chicago, IL) with significance accepted at *P* ≤ 0.05.

## 3. Results

### 3.1. CD4+

**Figure 2:**
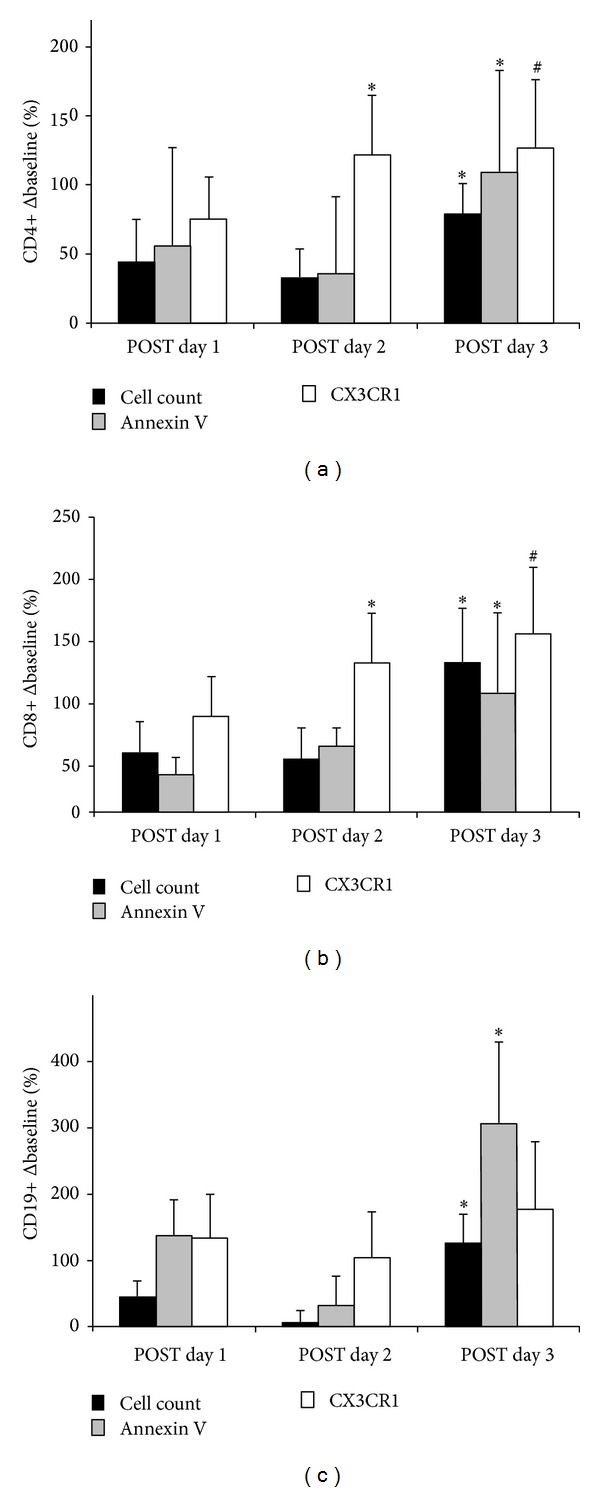
Absolute change from baseline in B lymphocytes CD4++ (a), CD8+ (b), and CD19+ (c) obtained from subjects (*N* = 12) following the treadmill bouts (day 1, day 2, and day 3). Data is for cell volume, apoptosis (annexin V+), and cellular migration (CX3CR1). * Significantly greater compared to the change in cell volume (*P* < 0.05).   ^∧^ Significantly less compared to the change in cell volume (*P* < 0.05).  ^†^ Significantly greater than previous day(s) (*P* < 0.05).

No differences were noted in CD4+ lymphocytes following the first or second day of interval running. Following the third consecutive day of interval running, the percentage change in all measures significantly increased compared to previous days of exercise (*P* < 0.05; see Figure 2(a)). In addition, the change in CD4+ cells marked for migration (CX3CR1) was significantly greater than the change in cell volume (*P* < 0.05; see Figure 2(a)).

### 3.2. CD8+

CD8+ lymphocytes were significantly modulated following the first day of interval running, such that there was significantly increased migration observed (*P* < 0.05; see Figure 2(b)) and a concurrent decrease in cells marked for apoptosis (*P* < 0.05). Following the second and third consecutive days of interval running, significant increases were noted in cells marked for migration (CD8+/CX_3_XR1, *P* < 0.05; see Figure 2(b)). Similar to what was observed in CD4+ lymphocytes, after the third consecutive day of interval running, the percentage change in all measures significantly increased compared to previous days of exercise (*P* < 0.05).

### 3.3. CD19+

With regard to CD19+ lymphocytes, significant increases were noted for both apoptosis and migration after the first and second consecutive days of intermittent running (*P* < 0.05; see Figure 2(c)). Furthermore, the increase in apoptosis was observed following the third day of interval running (*P* < 0.05). After the third consecutive day of treadmill running, the CD19+ cell volume response was significantly greater than the previous two days (*P* < 0.05).

## 4. Discussion

The main purpose of the present study was to investigate the effects of three consecutive days of high-intensity interval running on CD4+ (helper), CD8+ (cytotoxic), and CD19+ lymphocyte cell markers of apoptosis and migration in untrained individuals. The novel finding of the present investigation is that repeated intense interval exercise affects CD4+, CD8+, and CD19+ lymphocytes differently with regard to apoptosis and migration after three consecutive days of intermittent run exercise to exhaustion. Helper T lymphocytes were the least responsive to intermittent exercise, with changes only being observed following the third consecutive day of running. Cellular migration was observed to have the greatest influence on cytotoxic T lymphocytes following each day of the interval runs. On the other hand, B-cells displayed increases in both apoptotic and migratory markers following the first two days of intermittent running, and the apoptotic response persisted following day three.

The inclusion of HIT as part of an exercise program could offer a more time-efficient approach to achieve specific performance and health goals. Furthermore, parameters such as time to exhaustion, time trial performance, VO_2_ peak, both maximal and submaximal running speeds, running economy, metabolic profile, and insulin sensitivity are improved in both trained and untrained individuals [18, 19]. Individuals performing HIT complete brief periods of high-intensity aerobic exercise (typically >90% VO_2max_) separated by recovery periods of lower-intensity aerobic exercise or rest [2]. Previous investigations have reported that running at either an intensity equal to maximal oxygen uptake or at maximal running velocity (V_max⁡_) may lead to superior chronic adaptations when compared to continuous running in high-level athletes [3] or elicit rapid improvement in “aerobic fitness” in recreational athletes [4]. In addition, maximal velocity running has been shown to assist in controlling blood pressure and improving endothelial function, among other health benefits [5, 6].

To the best of our knowledge, this is the first study to investigate cellular markers of apoptosis and migration following three days of consecutive high-intensity interval treadmill runs in different lymphocytes' subsets. Previous investigations have investigated cell markers of apoptosis and migration following a progressive treadmill protocol to exhaustion [20], an endurance run on the treadmill for 2 h at 65% VO_2max_ [21], a treadmill protocol increasing intensity [10], repeated Wingate cycle tests [22], and a downhill running protocol [23]. Recently, Navalta et al. [10] assessed the lymphocyte subset response to increasing intensity (10 min run at 76% VO_2max_, 5 min at 87%, and run to exhaustion at 100% intensity). Cell concentration, apoptosis (annexin V), and migration (CX 3 CR1) were evaluated in CD4+, CD8+, and CD19+ subsets at rest, following each intensity, and 1 h later. The authors showed that subsets respond differently with intensity with respect to cell count and markers of apoptosis and cell migration. CD4+ and CD8+ appear to be prone to apoptosis with moderate exercise. In the present study, changes in T lymphocytes (CD4+, CD8+) were influenced to a greater extent by cellular migration. In contrast, an increased apoptotic influence was evidenced in B lymphocytes throughout the three consecutive days of interval running to exhaustion. It is likely that the combination of intensity, duration, and frequency in the present investigation accounts for differences to previously published reports.

One other investigation including leukocyte apoptosis utilized three consecutive days of treadmill running; however, a constant workload was applied. Tuan et al. [13] showed that plasma concentrations of TNF-*α* and sFasL were significantly raised after a short term, during three consecutive days of high-intensity exercise (85% of VO_2max_ for 30 min every day). Moreover, leucocyte mitochondrial transmembrane potential decreased significantly and immediately after each treadmill session, and this was accompanied by a substantial increase in apoptosis of peripheral blood leucocytes. These findings are similar compared to the present investigation and likely due to the exercise protocol used (i.e., three intense consecutives days of running). However, they used trained male runners (VO_2max_ of 70.4 mL·kg^−1^·min^−1^) and in the present study we used physically active volunteers.

There are some limitations to this study that should be noted. First, the subjects were recreationally active. Whether the results can be applied to well-trained subjects remains unclear. Second, the study did not include a control group, and third dietetic control was not taken into consideration. Moreover, additional time points after exercise would be interesting to analyze the recovery of the immune system following three consecutive days of high-intensity interval running.

In summary, our results provide evidence that repeated intense interval exercise appears to affect CD4+, CD8+, and CD19+ differently with regard to cell death and migration after the third day of running. In this sense, considering that the acute response to intense interval exercise in CD4+, CD8+, and CD19+ lymphocytes is magnified concerning migration and apoptosis, a rest day incorporated after two consecutive days of high-intensity intermittent running could minimize the effect on lymphocyte modulations and reduce the potential susceptibility to antigens during this timeframe.
